# Application of whole genome and RNA sequencing to investigate the genomic landscape of common variable immunodeficiency disorders

**DOI:** 10.1016/j.clim.2015.05.020

**Published:** 2015-06-26

**Authors:** Pauline A. van Schouwenburg, Emma E. Davenport, Anne-Kathrin Kienzler, Ishita Marwah, Benjamin Wright, Mary Lucas, Tomas Malinauskas, Hilary C. Martin, WGS500 Consortium, Helen E. Lockstone, Jean-Baptiste Cazier, Helen M. Chapel, Julian C. Knight, Smita Y. Patel

**Affiliations:** a Nuffield Department of Medicine, University of Oxford, Oxford, UK; b Oxford NIHR Biomedical Research Centre, John Radcliffe Hospital, Oxford, UK; c Wellcome Trust Centre for Human Genetics, University of Oxford, Oxford, UK; d Cold Spring Harbor Laboratory, W. M. Keck Structural Biology Laboratory, Cold Spring Harbor, NY 11724, USA; e Centre for Computational Biology, University of Birmingham, Haworth Building, B15 2TT Edgbaston, UK

**Keywords:** Common variable immunodeficiency, Whole genome sequencing, Transcriptome, Polygenic, B-cell

## Abstract

Common Variable Immunodeficiency Disorders (CVIDs) are the most prevalent cause of primary antibody failure. CVIDs are highly variable and a genetic causes have been identified in <5% of patients. Here, we performed whole genome sequencing (WGS) of 34 CVID patients (94% sporadic) and combined them with transcriptomic profiling (RNA-sequencing of B cells) from three patients and three healthy controls. We identified variants in CVID disease genes *TNFRSF13B, TNFRSF13C, LRBA* and *NLRP12* and enrichment of variants in known and novel disease pathways. The pathways identified include B-cell receptor signalling, non-homologous end-joining, regulation of apoptosis, T cell regulation and ICOS signalling. Our data confirm the polygenic nature of CVID and suggest individual-specific aetiologies in many cases. Together our data show that WGS in combination with RNA-sequencing allows for a better understanding of CVIDs and the identification of novel disease associated pathways.

## 1. Introduction

Common Variable Immunodeficiency Disorders (CVIDs) are the most clinically prevalent primary antibody deficiencies, present in about 1 in 25,000 people [1]. CVID is characterised by recurrent infections, low serum levels of IgG, low IgA and/or IgM and poor specific antibody responses [2]. CVID is currently a diagnosis of exclusion leaving a highly heterogeneous patient group in terms of clinical features and complications. Some patients have a relatively mild phenotype, comprising of recurrent bacterial infections and infection-related complications while others suffer from disease related complications such as autoimmune cytopenias, polyclonal lymphoproliferation, enteropathy and lymphoid malignancy, indicating underlying immune dysregulation [3].

The heterogeneous disease phenotype of CVID has made resolving genetic aetiologies challenging. Causative variants of CVID-like primary antibody failure have been described in *CD19* [4–6], *CD21* [7], *CD81* [8], *CD20* [9], *ICOS* [10,11] and *TNFRSF13C* [12], conditions now classified as specific deficiencies in these genes (Table S1). Mutations in *CD27* [13, 14], *PLCG2* [15,16], *LRBA* [17,18], *NFKB2* [19,20], *PIK3CD* [21,22] and *NLRP12* [23] cause CVID-like symptoms often combined with a more extensive clinical phenotype (Table S1). Variants in *TNFRSF13B* [24–26], *TNFRSF13C* [27], *FCGR2A* [28] and HLA [29] have been described to predispose to CVID (Table S1). Together these variants only explain the genetic cause of CVID-like diseases in very few patients and all genes were identified in familial cases of CVID, while the vast majority of CVID patients are sporadic. The wide variety in genes implicated in CVID further underlines the heterogenic nature of the disease. Further unravelling of the underlying genetic causes of sporadic CVID would give additional insight into the disease, opportunities for better patient stratification and novel insights into treatment opportunities.

In 2011 Orange et al. published the first genome-wide association study (GWAS) of CVID to identify genomic regions associated with CVID development [30]. Analysis of 363 patients and 3031 controls led to the conclusion that CVID is likely to be a polygenic disease with multiple novel susceptibility loci implicated. However, as of yet this has not resulted in further identification and elucidation of genes or variants that cause or predispose for sporadic CVID emphasizing the difficulties in studying this highly variable disease.

The development of next generation sequencing techniques has transformed the identification of the genetic basis of Mendelian diseases. In contrast, identification of the genetic basis remains challenging in polygenic conditions. Here, we present the first whole genome sequencing (WGS) data for a cohort of CVID patients to investigate novel underlying aetiologies. We further leveraged the potential of WGS by combining the results with global transcriptomic profiling through RNA-sequencing (RNA-seq). Because of the complex and probable polygenic nature of CVID, we combine the identification of genes of interest with pathway-based analysis and focus on combining these results to identify pathways dysregulated in CVID.

## 2. Material and methods

### 2.1. Samples

Patients were recruited into the study through the Clinical Immunology Department at the Oxford University Hospital, Oxford. All patients gave informed written consent and the studies were performed according to the Declaration of Helsinki. All 34 patients were of Caucasian origin and met the ESID diagnostic criteria at the time of enrollment [2]. The majority of patients were regularly followed in the Clinical Immunology clinic at 6 monthly intervals over a period of up to 30 years with detailed clinical information entered into the local database that enabled accurate clinical phenotyping. A summary of the clinical phenotype and laboratory characteristics of the patient cohort can be found in Table 1 and a more complete overview can be found in Table S2.

### 2.2. WGS500

A cohort of 34 CVID patients was selected for WGS as part of the WGS500 project [31]. This is a collaborative project between the University of Oxford and Illumina, which aims to sequence the genomes of 500 individuals with a range of diseases including rare inherited diseases, immunological disorders and cancer. For all variants the frequency of the variant in the non-cancer, non-CVID samples of the WGS500 project (n = 239) is listed in Table 2 and Tables S3–S7 and S15.

### 2.3. Whole genome sequencing

Genomic DNA was extracted from peripheral blood mononuclear cells (PBMCs) using the FlexiGene DNA kit (Qiagen) according to the manufacturer’s instructions. The DNA was quantified by fluorescence using the Quibit Fluorometer (Invitrogen) and the quality assessed by running <500 ng on a 1% TAE agarose gel for 1 h at 70 V. Whole genome sequencing was performed on 3.5–7.5 ng DNA on either the Illumina HiSeq2000 or the HiSeq2500 run in standard mode using v2.5 or v3 sequencing chemistry (Core Genomics, WTCHG). Briefly, the genomic DNA was fragmented, end-paired, A-tailed and adapter-ligated before size selection and amplification for a multiplexed library preparation. The libraries were then paired end sequenced and sequenced to an average coverage between 27 and 40 quality reads per base (100 base pair reads).

### 2.4. Mapping and variant calling

Reads were mapped to the GRCh37d5/hg19 human reference sequence using Stampy and the SNVs and short InDels were called with Platypus www.well.ox.ac.uk/platypus (Core Bioinformatics, WTCHG) [32].

### 2.5. Variant filtering

The created vcf files were analysed using QIAGEN’s Ingenuity® Variant Analysis^™^ (IVA) software (www.qiagen.com/Ingenuity, QIAGEN Redwood City). For all analysis a confidence filter was applied to exclude poor quality calls (Table 3) [33]. Frequency (1000 Genomes and WGS500) and predicted deleterious filters (SIFT and PolyPhen) were applied to prioritise high quality calls for non-synonymous and likely pathogenic variants (Table 3). IVA allowed the use of custom gene lists (Notes S1 and S3) as biological filters with options for ‘1 hop’ analysis both up and downstream of these genes [34]. The ‘1 hop’ function identifies interactions between genes from literature. Here, this function was used to create the list of variants in the genes interacting with described CVID genes.

Genomic regions described in GWAS were transferred from hg18 into hg19 using http://www.genome.ucsc.edu/cgi-bin/hgLiftOver. WGS variants were filtered as described in Table 3 and restricted to those in the identified genomic regions [30].

All variants reported (except the results of the pathway analysis) were manually inspected using the Integrative Genome Viewer (IGV) [35]. Heterozygous variants were included if the variant was present in >25% of all reads with >15% in both forward and reverse reads. Homozygous variants were included if present in >80% of all reads and a minimum of 60% of reads in both directions. Variants found in regions with a high density of insertions/deletions were excluded to reduce false positive calls resulting from mapping errors.

### 2.6. Sanger sequencing

Primers were designed using primer3 software (http://bioinfo.ut.ee/primer3/) (Table S14). 25 ng DNA was amplified using a 25 μl PCR reaction containing 2.5 μl 10× Buffer, 1.5 mM MgCl_2_, 0.2 mM dNTP, 250 nM primer (each direction) and 0.5 U Platinum Taq DNA polymerase (Invitrogen). Cycling conditions were an initial denaturation step at 94 °C for 2–5 min followed by 30 cycles of 94 °C for 30 s, 56–63 °C (Table S14) for 30 s and 72 °C for 40 s followed by a final elongation step of 5 min at 72 °C. The PCR product was cleaned using either a QIAquick PCR purification kit (Qiagen) or by incubating 0.1 μl ExoI and 0.1 μl CIP with 0.4 μl NEB buffer 3 in an 8 μl reaction at 37 °C for 30 min before inactivation at 95 °C for 5 min. 3.5 μl cleaned-up template DNA was sequenced using the Big Dye Terminator 3.1 according to the manufacturer’s instructions (Applied Biosystems).

### 2.7. RNA-sequencing

RNA-seq data were generated for enriched B cells from three CVID patients and three healthy controls. PBMCs were isolated using Lymphoprep (Stemcell Technologies) and B-cells were enriched using CD19 microbeads (Miltenyi Biotec) according to the manufacturer’s instructions. After isolation, purity of the B-cells was 61.9–96.1% as determined by FACS analysis. RNA was extracted using a RNeasy Mini Kit (Qiagen) following the manufacturer’s instructions. Spectrophotometry (Nanodrop 2000; Thermo Scientific) was used to quantitate the RNA yield and the quality was determined by on-chip electrophoresis (Biorad Bioanalyzer; Agilent). RNA-seq was performed on 600–1000 ng RNA on the Illumina HiSeq2500 run in standard mode using v1 sequencing chemistry (Core Genomics, WTCHG). Briefly, the mRNA fraction was selected from the total RNA before conversion to cDNA. dUTP was incorporated from the second strand cDNA synthesis. The cDNA was end-repaired, A-tailed and adapter-ligated. Prior to amplification the samples underwent uridine digestion. The prepared libraries were size selected, multiplexed and QC’ed before paired end sequencing over one lane of a flow cell. Data were aligned to the reference and reads mapped to genes using HTSeq (Core Bioinformatics, WTCHG) [36]. QC to determine the proportion of reads mapping to genes and variation within the dataset was carried out using R (R Core Team, 2012; http://www.R-project.org) (see Fig. S4). The genes with mapped reads were filtered so only those with at least 10 reads in at least three samples were retained prior to normalisation. This resulted in a reduction from 32,426 to 16,546 transcripts. The data were normalised using the TMM method and the edgeR R package [37]. Biological pathway enrichment was generated through the use of QIAGEN’S Ingenuity Pathway Analysis (IPA®, www.qiagen.com/ingenuity, QIAGEN Redwood City).

### 2.8. Protein structural analysis

Variants were mapped to corresponding protein structures in Protein Data Bank (PDB) (LRBA, Protein Data Bank (PDB) ID 1T77; TNFAIP3, PDB ID 3ZJE; SH3BP2, PDB ID 2CR4) (http://www.rcsb.org/) [38]. The structures were analysed using PyMOL (The PyMOL Molecular Graphics System, Version 1.5.0.4 Schrödinger, LLC; http://pymol.org/). The effect of a variant on protein stability was assessed using mCSM [39].

## 3. Results

### 3.1. Whole genome-sequencing and CVID

As part of the WGS500 project aiming to sequence the genomes of 500 individuals with a range of diseases, we performed WGS for a cohort of 32 sporadic CVID patients and one grandmother–grandson pair [31]. The cohort had an equal sex distribution and the median age of disease onset was 23.5 years. All patients met the European Society for Immunodeficiencies (ESID) diagnostic criteria for CVIDs [2]. We excluded patients with lymphomas, though patient C003 developed a B cell lymphoma four years into the study. Information on the clinical phenotypes and immunotypes of the patients can be found in Table 1 and Table S2. WGS using the Illumina platform with an average coverage between 27 and 40 quality reads per base identified 14,819,871 variants (single nucleotide variants (SNVs) and short InDels) across the 34 patients. Initial analysis confirmed a familial relationship between the grandmother (D232) and grandson (C018) pair and revealed long runs of homozygosity in one of the sporadic patients (D269).

To validate our data we first looked for variants previously described to cause or predispose for CVID-like diseases in our patients (variants are listed in Table S1, results are summarised in Table S3). This analysis revealed five previously reported variants in *TNFRSF13B*, tumour necrosis factor receptor superfamily member 13B (also known as TACI). We identified one patient heterozygous for p.C104R, previously reported to abolish ligand binding in the heterozygous state through haploinsufficiency [24,26,40]. Other *TNFRSF13B* variants (p.P251L, p.V220A, p.R202H and p.R72H) have originally been linked to CVID, but a later study found equal frequencies in CVID patients and healthy controls as we found here (as compared to the frequency in the 1000 genomes project and the non-CVID WGS500 samples) [24–26,41]. Our analysis also identified one patient heterozygous for the p.H304Y variant in the inflammatory modulator gene *NLRP12* [23]. We identified two patients with the p.P21R variant (one homozygous) in tumour necrosis factor receptor superfamily member 13C gene *TNFRSF13C* (BAFFR) [27] which is reported to have functional consequences through effects on multimerisation [42]. We noted poor coverage of *TNFRSF13C* in our WGS data and subsequent Sanger sequencing of all 34 patients revealed an additional 4 patients heterozygous for this variant (frequency of 0.176 compared to 0.059 in the 1000 Genomes Project) [41]. Details of variants highlighted throughout this report can be found in Table 2.

**Fig. 1:**
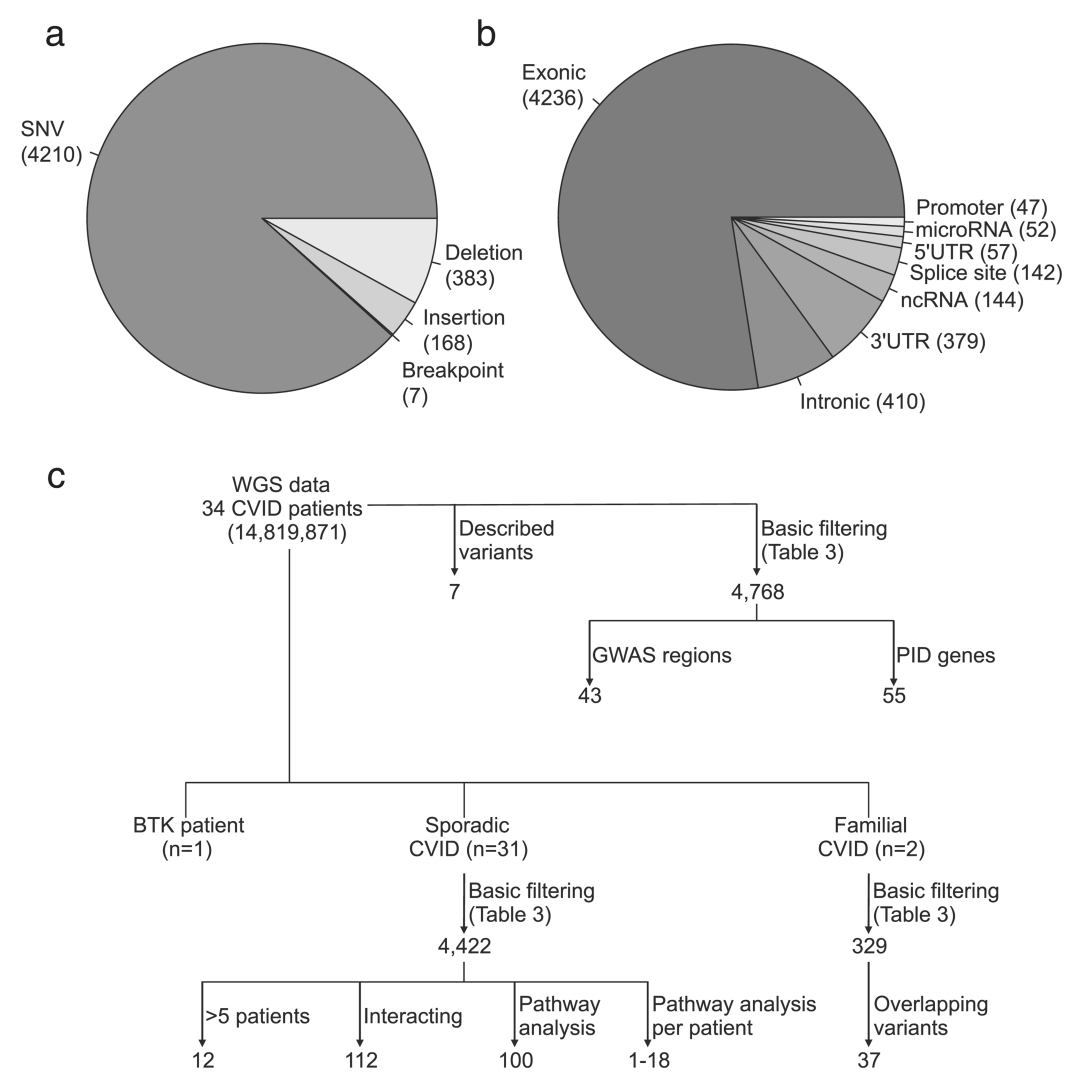
Overview of analysis strategy and distribution of variant type and genetic location of variants. (a) Distribution of variant type for variants that passed the filtering steps as described in Table 3. (b) Number of variants in different genetic locations in the 4768 variants that passed the basic filtering described in Table 3. (c) Overview of the analysis strategies applied to the WGS data.

To identify novel variants of interest we selected high quality, and likely pathogenic variants. Because of the expected polygenic nature of CVID we used the relatively mild selection criteria (1000 Genomes and WGS500 frequency <0.05 and classified as pathogenic or likely pathogenic by Ingenuity Variant Analysis (IVA)), and included both heterozygous and homozygous variants resulting in 4768 variants in 3737 genes (details on the filtering can be found in Table 3). The majority of variants were SNVs (Fig. 1a) in exonic regions together with a number in annotated regulatory regions including promoters, 5′ and 3′UTRs, microRNA binding sites, splice sites and noncoding RNAs (Fig. 1b). In our analysis strategy (Fig. 1c) we focused on variants present in regions implicated in GWAS [30], genes involved in described primary immunodeficiency disorders (PIDs) or genes with a direct link with previously identified CVID genes. Moreover, we also looked at pathways in which variants were overrepresented.

### 3.2. GWAS and PID genes

The CVID GWAS conducted by Orange and colleagues [30] included 29 of the patients described here. Within the regions associated with CVID development we identified 43 potentially pathogenic rare variants (Table S4) of which 39 are located in the HLA region of chromosome 6. We highlight the variants of interest based on a combination of gene function, frequency (1000 Genomes Project and WGS500 compared to our cohort) and predicted deleteriousness (PolyPhen, SIFT and mutation taster prediction score) (Table 2) [41,43–45]. Two variants were identified in *SKIV2L*, a gene associated with trichohepatoenteric syndrome 2 and syndromic diarrhoea, two rare diseases associated with low immunoglobulin levels and the absence of vaccine responses [46]. Four variants (p.R1883Q, p.P1745R, p.R1299Q, p.S1112F) were observed in *MDC1*, a gene involved in DNA damage response [47]. We also identified six patients (17.6%) with predicted deleterious variants in *PRRC2A* (p.R804C, p.R1556W, p.R1397W, p.F2083S) a gene associated with type 1 diabetes and rheumatoid arthritis, suggesting a function in immune regulation [48,49]. We did not find any predicted deleterious variants in *CLEC16A*, a gene recently associated with CVID via GWAS [50].

Next, we hypothesised that a combination of heterozygous variants in genes where homozygous variants cause PIDs (Note S1) may contribute to the pathogenicity of CVID as previously described for rheumatoid arthritis [51]. Analysis resolved 55 variants in described PID genes (Table S5). Again the most likely disease causing variants were identified based on frequency, gene function and predicted deleteriousness (Table 2). Variants of interest include a novel, predicted damaging p.S2221P/p.S2210P variant in *LRBA* and heterozygous predicted deleterious variants in other genes in which loss of function leads to low or absent immunoglobulin levels (*IKBKB*), combined with low or absent B-cell subtypes (*TCF3, BTK, CD79A, PMS2, POLE*), and/or T-cell dysregulation (*IL21R, STIM1, TAP2, DKC1*) [52].

We identified five CVID patients with heterozygous variants in genes which are involved in non-homologous end joining (NHEJ) or V(D)J recombination and associated with T–B-SCID (*DCLRE1C, PRKDC, RAG2*) [52,53]. We found an additional four variants in other PID genes involved in NHEJ (*NHEJ1, MRE11A, ATM, NLRP2*) [53]. We also identified variants in PID genes involved in B-cell receptor (BCR) signalling (*IKBKB, CD79a, BTK, KRAS*) including a novel hemizygous variant in *BTK* (p.N350fs11/p.N526fs11/p.N560fs11) that leads to a frameshift and a premature stop codon. This male patient was previously screened for known *BTK* mutations and clinically fits the phenotype of X-linked agammaglobulinaemia (XLA) caused by BTK deficiency [52]. Functional follow-up is currently being conducted on this finding.

### 3.3. Genes in sporadic CVID

Next, we further investigated the 31 sporadic CVID patients in our cohort (excluding the grandmother:grandson pair (Note S2) and the possible XLA patient). Filtering as described in Table 3 identified 4422 variants of which 12 were present in five or more patients (Table S6). Of special interest is a likely gain of function variant in *LILRB5*, an inhibitory member of the leukocyte Ig-like receptor (LIR) family, of which little is known though other members of this protein family inhibit the activation of immune cells (Table 2) [54].

Likely pathogenic variants in genes for which there is evidence from the literature of direct interaction with genes involved in CVID-like antibody failure were investigated using the ‘1 hop’ function in IVA (Note S3). We identified 112 variants (Table S7) of which 38 were novel, including predicted damaging variants in *PSMB9*, a gene involved in the production of peptides for MHC class I presentation, and *TNFAIP3* which plays a role in TNFα induced apoptosis and at the protein level is known to interact with *TNIP1* in which we identified two different novel variants [55]. In addition we found variants in seven genes involved in B-cell receptor signalling (*ARID3A, INPP5D, SH3BP2, BANK1, BLK*, and *GAB2*), a variant in *CAMLG* encoding a protein involved in both TACI and TCR signalling, and a variant in *FCGR3B* encoding FCγR3B [56–62]. One patient (C032) was found to have a variant in the 3′UTR regions of two genes (*BCL2L11* and *EBF1*) involved in IL7Rα signalling [63,64]. Dysregulation of this pathway leads to low T-cell numbers and low antibody production [52]. Moreover, *EBF1* is a master regulator of early B-cell development in the bone marrow [63,65].

**Fig. 2:**
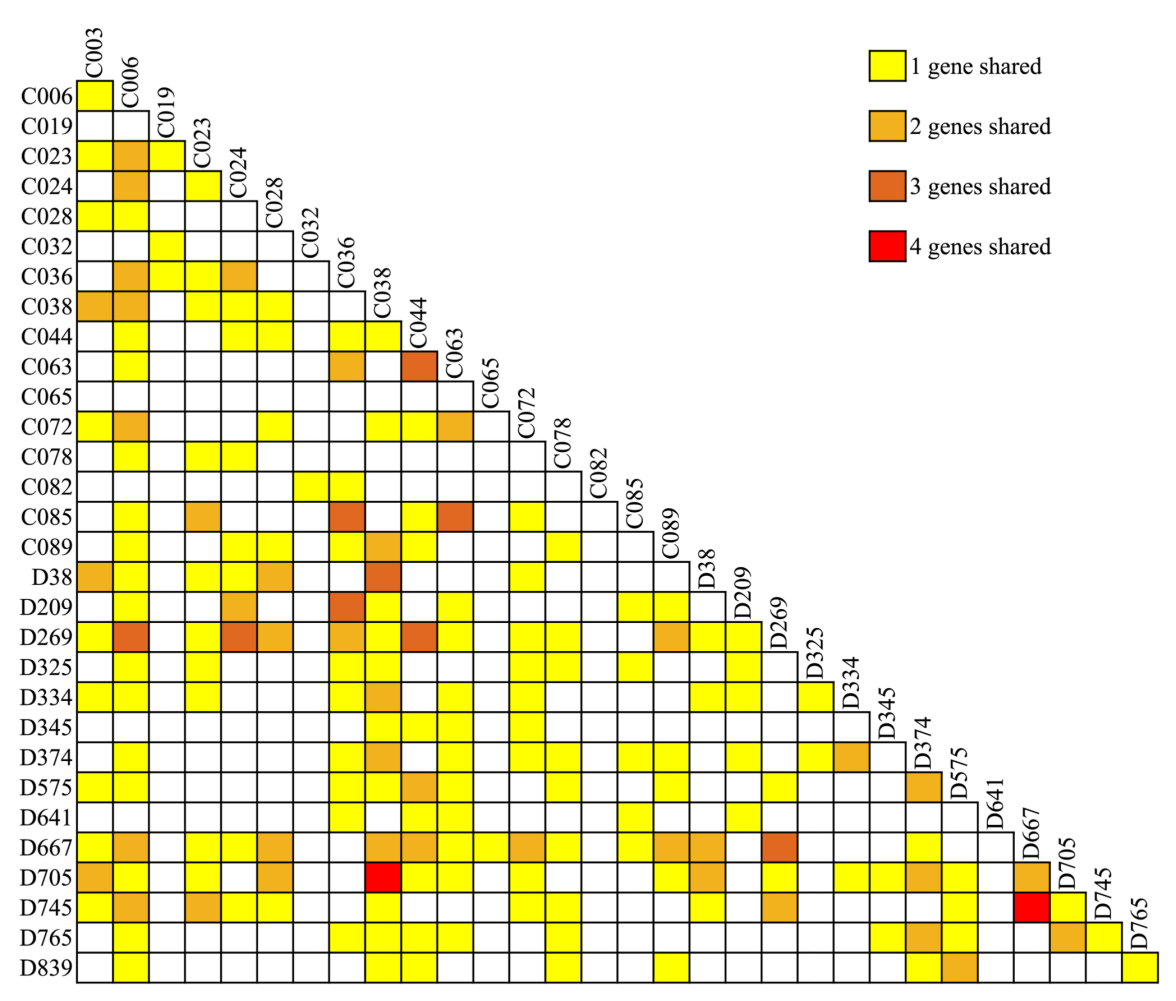
Distribution of variant-containing genes between patients. The number of variant-containing genes from Tables S3–S7 shared between patients.

**Fig. 3:**
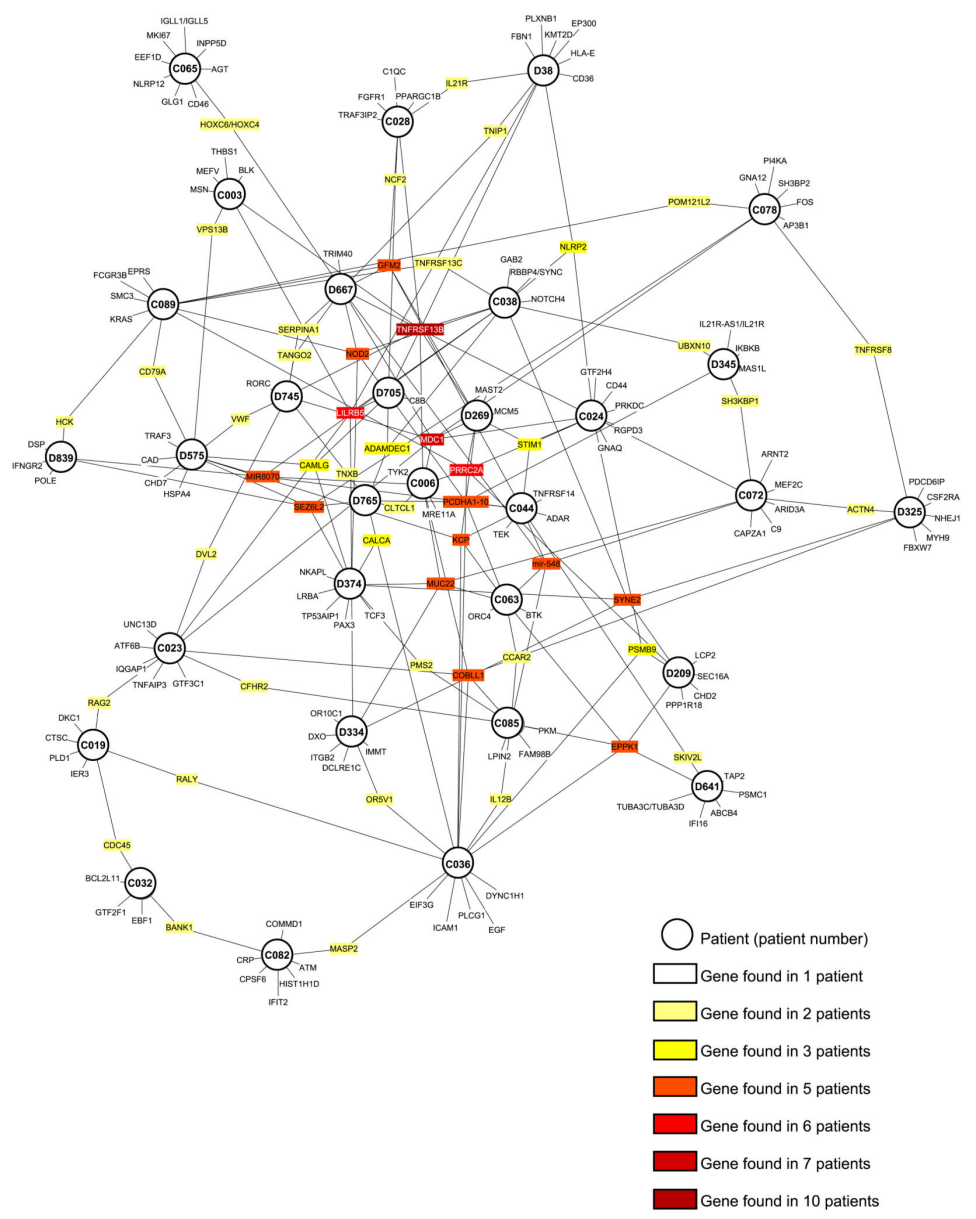
Visualisation of the overlap of variant containing genes between patients. The distribution of variant-containing genes (small nodes) listed in Tables S3–S7 between patients (big nodes).

Overall, our analysis strategy to identify variants associated with CVID, variants in GWAS regions, PID genes, the most common variants and variants in genes interacting with described CVID genes (Fig. 1c), identified on average 9.4 variants (range 5–15) in each of the 31 sporadic CVID patients. Two patients do not share any variants with the other patients, while in others, the majority (up to 87%) of variants are also found in one or more other patients (Fig. S1 and S2). A maximum of five variants are shared between two patients. Fig. 2 shows the overlap of variant-containing genes between patients. Visualising this shows that all patients share at least one variant-containing gene with another patient (Fig. 3). Patients did not cluster together based on their clinical phenotype (IgM+/− and complex vs infections only phenotypes) (Fig S3). We did not have sufficient power to do a comparison analysis between different patient groups.

### 3.4. Integration with RNA sequencing analysis

**Fig. 4:**
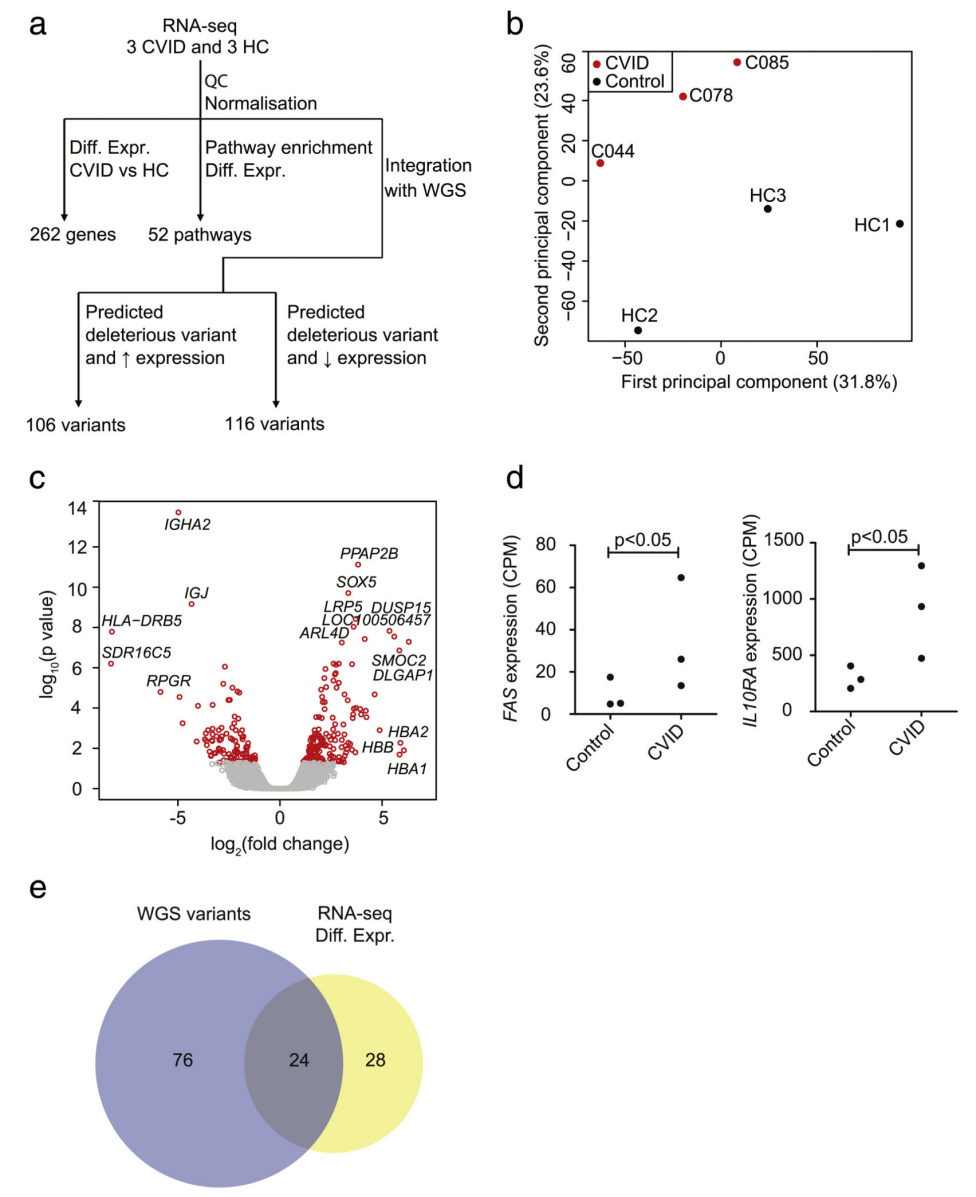
Overview of differentially expressed genes and overlap of pathway analysis of WGS and RNA-seq data. (a) Overview of the analysis strategy applied to the RNA-seq data. QC, quality control; Diff. Expr., differentially expressed; HC, healthy controls. (b) The first two principal components are plotted with the proportion of variance explained by each component (based on reads which map to genes). (c) Volcano plot of differentially expressed genes. The significance of differential expression between CVID patients and healthy controls is plotted against fold change. Each point represents a single gene. Red points highlight genes with an adjusted p value < 0.05 and fold change >2. A positive fold change indicates upregulation in the CVID patients. Some of the most differentially expressed genes are named. (d) Differential expression of *FAS* and *IL10RA* between CVID patients and healthy controls. (e) The 100 most significantly enriched pathways for WGS predicted deleterious variants were reported by IVA. Fifty-two pathways were enriched for differentially expressed genes from the RNA-seq analysis. The overlap of enriched pathways between these two datasets is greater than expected by chance (Fisher’s exact test p value < 0.0001).

As a pilot study, RNA-seq data were generated for B-cell enriched samples from three sporadic CVID patients (C078, C085 and C044, selected on B-cell numbers and sample availability) and three healthy controls (Figs. 4a–b; Fig. S4). We performed differential gene expression analysis between CVID patients and healthy controls for 16,546 transcripts revealing 262 transcripts that were differentially expressed (false discovery rate <0.05 and fold change >2) (Fig. 4c; Table S8). Down-regulated genes included those encoding immunoglobulin heavy and light chains as would be expected. This, together with our ability to reproduce a previous study showing increased *FAS* (a member of the TNF receptor superfamily encoding a receptor inducing programmed cell death) expression in CVID patients suggests that this small pilot study is able to pick up important differences in gene expression (Fig. 4d) [66]. We also find significantly increased expression of four additional PID-associated genes in the CVID patients (Fig. 4d and Fig. S) including *IL10RA*, encoding the receptor for interleukin 10 that mediates immunosuppressive signals. None of the genes located in regions implicated by GWAS or genes highlighted in previous paragraphs (Table 2) were significantly differentially expressed.

The WGS analysis we performed enables the prediction of variants deleterious to protein function. Combining WGS with RNA-seq allowed us to identify non-coding variants with evidence of altered gene expression. For each of the three patients with RNA-seq data, we applied filters to their WGS data as described in Table 3, with an alteration to the predicted deleterious filter to include variants which modify promoter and enhancer regions in addition to those with predicted deleterious effects to protein function. To determine gain of function variants we identified variants linked to genes with higher expression in CVID patients compared to healthy controls (fold change >2 from median expression) (n = 106, chi squared p value < 0.0001, Table S9). Fig. S6 shows the increased expression of *ITGB2*, a described PID gene, and *ICAM1*, which interacts with three CVID related genes (*NLRP12, CD81, PLCG2*). We also identified variants linked to reduced gene expression (n = 116, chi squared p value < 0.0001, Table S10). These include *SH3BP2* which interacts with and activates other CVID and PID genes and *VPREB3* which is involved in B-cell development (Fig. S6) [67,68]. No variants linked to altered gene expression are found in the regions associated with CVID identified by GWAS [30].

### 3.5. Pathway analysis using WGS and RNA-seq

Due to the heterogeneous and probable polygenic nature of CVID we hypothesised that different variants or combinations of variants in the same pathway could lead to similar clinical phenotypes. Therefore we focused on the identification of pathways that are possibly dysregulated in CVID. We identified the 100 pathways most enriched for genes with predicted deleterious variants in the 31 sporadic CVID patients based on WGS data (Table S11) and compared this with the 52 pathways enriched for differentially expressed genes in the three CVID patients compared to healthy controls from Ingenuity Pathway Analysis (IPA) (Table S12). We found that 24 of these pathways overlap (Table 4), which is significantly greater than expected by chance (Fisher’s exact test p < 0.0001; Fig. 4e). The presence of ‘iCOS–iCOSL signalling’ in both analyses confirms previous findings [10,11]. Other pathways of interest include those involved in the development of the immune system (‘haematopoiesis from pluripotent stem cells’) and regulation of apoptosis (‘death receptor signalling’); and also pathways involved in cytokine signalling (‘IL-6 signalling’, ‘IL-10 signalling’) and differentiation or signalling of T helper cells (‘CD28 signalling’, ‘PKCθ signalling’ and ‘T helper cell differentiation’).

CVID may result from dysregulation of different pathways in different patients and analysing all 31 patients as a group may not reveal this. Therefore we repeated the WGS pathway analysis on a patient by patient basis to identify potentially important pathways at the individual level. This revealed an average of 5.8 (range 1–18) significantly enriched pathways per patient (Table S13). Pathways of particular interest include ‘PI3K/AKT signalling’ (three patients) and ‘the role of BRCA1 in DNA damage response’ (three patients) ‘Actin Cytoskeleton signalling’ (two patients) and ‘DNA Double-strand Break repair by non-homologous end joining’ (one patient).

### 3.6. Structural perspective on predicted deleterious variants

**Fig. 5:**
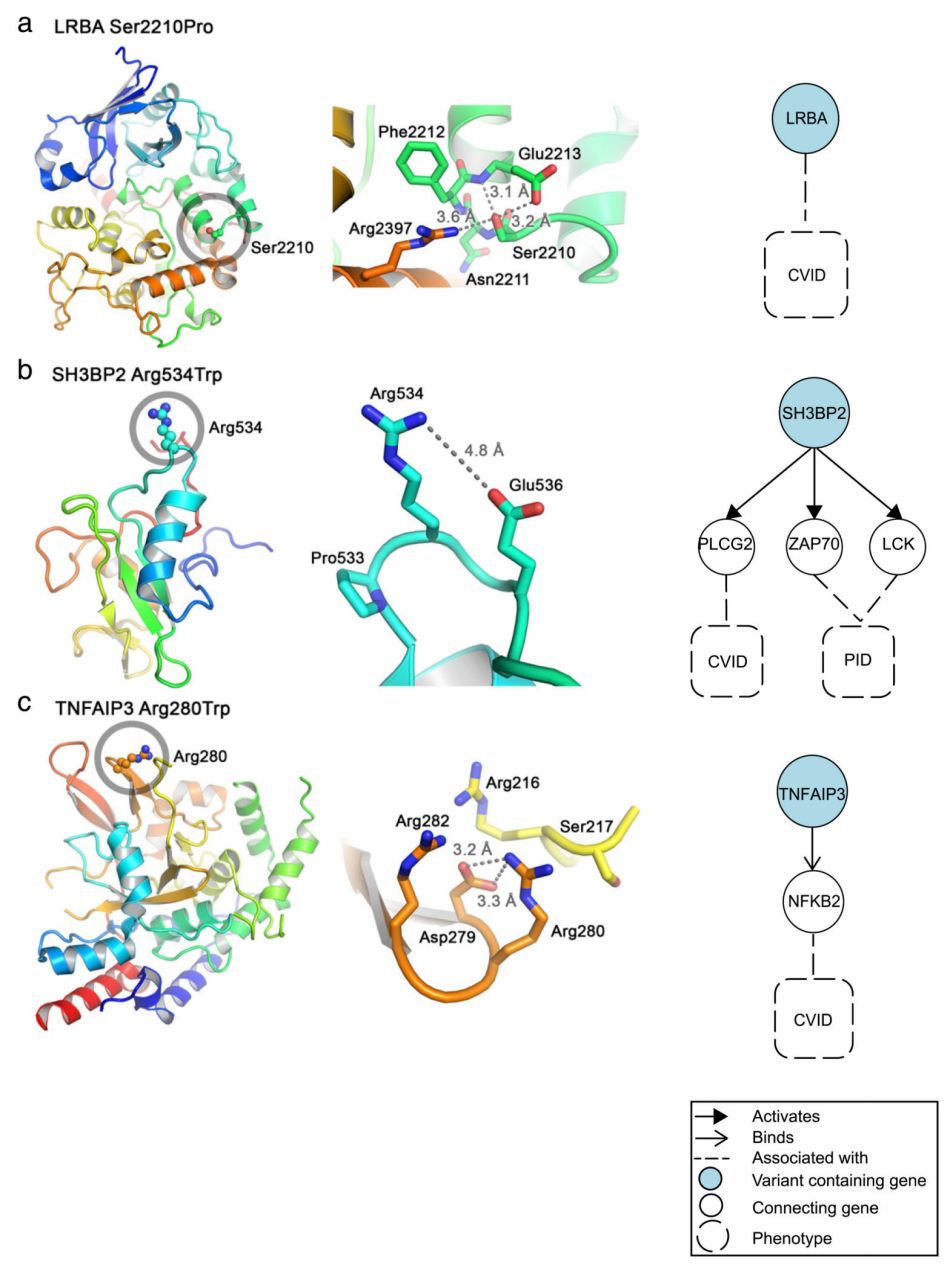
Mapping of predicted deleterious variants onto protein structures and linking proteins of interest to CVID. Proteins are shown as ribbon diagrams and coloured in blue-to-red transitions (N-termini, blue; C-termini, red). Side chains of selected variants are shown as connected spheres (oxygen, red; nitrogen, blue) and are circled in the panels on the left. Zoom-in views are presented in the panels on the middle with side chains shown as sticks. Distances between selected atoms are indicated with dashed lines. The right panel shows how these proteins could be linked to CVID. (a) The crystal structure of the C-terminal fragment of human LRBA encompassing the pleckstrin homology (PH, blue) and the beige and Chediak–Higashi syndrome (BEACH, green-to-red) domains (PDB code 1T77) [69]. The side chain of Ser2210 makes hydrogen bonds to Glu2213 (both main chain and side chain) and the side chain of Arg2397. Amino acid numbering corresponds to UniProt entry P50851, isoform 2. (b) Solution NMR structure of the SH2 domain of SH3BP2 (PDB 2CR4; UniProt P78314, isoform 4). The side chain of Arg534 is exposed on the protein surface and does not make any bonds with other residues. (c) The crystal structure of the catalytic domain of TNFAIP3 (PDB 3ZJE; UniProt P21580) [70]. The side chain of Arg280 is exposed on the surface and makes hydrogen bonds to the side chain of Asp279.

To further understand the mechanism by which the predicted deleterious variants (Table 2) could modulate protein structure and function, we mapped them onto available structures. Three selected variants highlighting changes of protein surface properties and interactions between secondary structure elements (helices) are shown in Fig. 5. Changes of protein surface properties could alter protein–protein interactions whereas disruption of bonding within the protein may alter folding, conformation and thus function. All three variants were mapped onto available crystal or solution NMR structures [69,70]. The novel variant p.S2210P that we identified in *LRBA* was found to affect an evolutionarily conserved, partially buried Ser2210 of LRBA located within the BEACH domain that is crucial for protein function [69]. The side chain of Ser2210 makes multiple hydrogen bonds with Glu2213 and Arg2397 (Fig. 5a). The Ser2210Pro mutation would abolish these interactions and could thus alter conformation and/or stability of LRBA. In contrast, with the variant p.R534W we identified in the B cell receptor signalling protein SH3BP2, the side chain of Arg534 does not interact with any residues within the SH2 domain (Fig. 5b). As Arg534 is exposed on the SH2 surface, the Arg534Trp mutation would make SH3BP2 more hydrophobic and could therefore alter SH3BP2 interactions with its potential binding partners recognising the surface of SH3BP2 centred on Arg534. The pR280W mutation of the deubiquitinase TNFAIP3 (also known as A20) involves Arg280 which is located within the catalytic domain and stabilizes conformation of the domain by binding to Asp279 (Fig. 5c) [70]. The Arg280Trp mutation would abolish these interactions and thus might affect the overall conformation, catalytic activity and/or TNFAIP3–protein interactions. We further assessed whether these three variants could affect protein stability using a programme mCSM [39]. All three mutations are predicted to destabilize the proteins (change in Gibbs free energy, ΔΔG, kcal/mol: LRBA Ser2210Pro, −0.236; SH3BP2 Arg534Trp, −0.461; TNFAIP3 Arg280Trp, −0.138).

## 4. Discussion

Here, we present the first WGS analysis of sporadic CVID patients and show that this does not reveal a unifying genetic aetiology in terms of highly penetrant mutations involving a single gene or pathway but rather that CVID is likely to include a number of different disease aetiologies matching a polygenic model. We applied an analysis strategy considering previously implicated candidate genes, loci implicated by CVID GWAS, together with known primary immunodeficiency disorder genes. Further investigation of genes with experimental evidence of interaction with those involved in CVID-like antibody failure was informative. We adopted an integrated analysis that considered WGS data in the context of differentially expressed genes in CVID patients, allowing interpretation in a biological context. We recognise that significantly larger sample sizes would allow further progress in resolving the heritable basis of CVID and resolving the current classification of disease aetiology. Our finding of a novel BTK mutation illustrates the diagnostic power of the WGS approach.

Among the CVID patients analysed, we found variants in *TNFRSF13B, TNFRSF13C* and *NLRP12*, consistent with previous findings [23–27]. We also found a novel heterozygous variant in *LRBA*, which is predicted to influence protein stability. Using our combined analysis strategy, we are able to identify a number of important pathways where we find evidence of pathogenic mutations which together with biological evidence that we and others have generated reveal likely significant pathways in CVID. For example we found significant enrichment in WGS and differential gene expression analysis of ICOS–ICOS ligand signalling confirming the previously found importance of this pathway in antibody failure [10,11].

Other studies have previously shown that deficiency in genes involved in B-cell receptor sequencing (*CD19, CD21, CD81, PLCG2, PI3KCD*) can lead to CVID-like antibody deficiencies [4–8,15,16]. In our analysis we found heterozygous coding variants in a variety of genes involved in this pathway (IKBKB, CD79a, BTK, KRAS, ARID3A, INPP5D, BANK1, BLK, and *GAB2*) including a number of novel variants in *BTK, BLK* and *ARID3A*. In one patient we found reduced expression and two variants (one coding and one non-coding) in *SH3BP2*. The coding variant might alter interactions with potential binding partners. Moreover, in our RNA-seq analysis BCR signalling was one of the most significantly enriched pathways in CVID patients.

Previous studies showed that variants in BAFF-R, a receptor important for B-cell survival, can cause or predispose to CVID [12,27,44]. We, and others, show increased expression of *FAS*, an apoptosis inducing receptor, in CVID patients [66]. Moreover, we found a variant which might alter the overall conformation of *TNFAIP3*, a gene inhibiting TNFα induced apoptosis, and novel variants in *TNIP1* which is a known *TNFAIP3* interaction partner. Death receptor signalling was significant in pathway analysis of both the RNA-seq and WGS analyses together suggesting that apoptosis might be dysregulated in a subgroup of CVID patients.

As NHEJ is essential in V(D)J recombination and class–switch recombination, inefficient NHEJ could lead to CVID like symptoms. We find variants in *DCLRE1C, PRKDC, RAG2, NHEJ1, MRE11A, ATM* and *NLRP2* in our patients, all genes important in the initiation of V(D)J recombination or DSB repair by NHEJ. The hypothesis that NHEJ defects could lead to CVID in a subgroup of patients is supported by the recent finding of a leaky block in B-cell development in a study comparing the bone marrow of CVID patients and healthy controls [71].

Our analysis also highlighted the role of T-cells in sporadic, non familial CVID. We found variants in genes associated with T-cell regulation (*IL21R, STIM1, TAP2*) with WGS. Pathway analysis of both the RNA-seq and WGS data revealed enrichment of a number of T-cell related pathways (T helper cell differentiation; CD28 signalling; ICOS–ICOSL signalling). Dysregulation of T cells could also be the result of pathways found to be significantly enriched in the WGS data related to dendritic cell maturation, interaction between innate and adaptive immune cells and cytokine signalling (IL-18, IL6 and IL10) which would lead to B cell dysfunction through altered B–T cell interactions in CVID patients.

Interestingly, we found a relatively high number of T-cell related pathways in our RNA-seq analysis, having investigated and compared the expression profiles of samples enriched for B-cells. One possible explanation is the presence of residual T-cells in our samples (61.9–96.1% B-cell purity). Alternatively, genes that are important in T-cell signalling pathways could also be expressed in B-cells and therefore be found in our analysis.

In one of our CVID patients we identified variants in the 3′UTR region of two genes involved in IL-7R signalling (*BCL2L11* and *EBF1*) [63,64]. 3′ UTRs are important for the determination of translation efficiency, localization and stability of mRNA. In mice IL-7R signalling and EBF-1 expression have been described as central regulators in B cell development where their interplay positively regulates B lineage commitment, survival and proliferation [64]. In humans, EBF-1 is important in B-cell lineage commitment [63]. In contrast, the role of IL-7R signalling in B-cell development seems less important as patients with IL-7Rα deficiency have normal B-cell numbers.

Together our data illustrate the strength of a combined approach using WGS and RNA-seq and highlight that CVID is a highly heterogeneous and polygenic disease. Our results corroborate with previous findings in *TNFRSF13B, TNFRSF13C* and *NLRP12*. Moreover, our data highlight variants in genes involved in ICOS–ICOSL signalling, regulation of apoptosis, and BCR signalling. We were also able to identify novel pathways which may be important for CVID, including NHEJ, T cell regulation and cytokine signalling. However, functional follow-up studies in individual patients are needed to confirm the role of variable pathways involved in the pathogenesis in CVIDs.

## Supplementary Material

Supplementary material

Supplementary tables

## Figures and Tables

**Table 1:** Overview of clinical information on the 34 CVID patients.

Patient no	Sex	Age at onset of symptoms	CD19 count^a^	CD3 count^a^	CD4 count^a^	CD8 count^a^	IgG levels^a^	IgM levels^a^	IgA levels^a^	Clinical phenotype^3^
Normal			0.1–0.5	0.7–2.1	0.3–1.4	0.2–0.9	6.0–16.0	0.4–2.5	0.8–3.0	
C003	F	54	↓ ↓	↓	Normal	Normal	↓ ↓	Normal	↓ ↓	Polyclonal lymphoproliferation
C006	M	34	Normal	Normal	Normal	Normal	↓ ↓	↓ ↓	↓ ↓	Polyclonal lymphoproliferation
C018	M	8	Normal	Normal	Normal	Normal	↓ ↓	↓ ↓	↓ ↓	No disease-related complications
C019	M	18	Normal	Normal	Normal	↑	↓ ↓	↓ ↓	↓ ↓	No disease-related complications
C023	M	25	Normal	Normal	Normal	Normal	↓ ↓	↓ ↓	↓ ↓	No disease-related complications
C024	F	48	Normal	↓	Normal	↓	↓ ↓	↓ ↓	↓ ↓	No disease-related complications
C028	F	47	Normal	Normal	Normal	Normal	↓ ↓	↓ ↓	↓ ↓	Polyclonal lymphoproliferation
C032	M	26	↓	Normal	↓	↓	↓	Normal	↓ ↓	Polyclonal lymphoproliferation
C036	F	19	Normal	Normal	Normal	Normal	↓ ↓	↓ ↓	↓ ↓	No disease-related complications
C038	M	5	Normal	Normal	Normal	Normal	↓ ↓	↓ ↓	↓ ↓	No disease-related complications
C044	F	31	Normal	Normal	Normal	Normal	↓	↓ ↓	↓	Polyclonal lymphoproliferation; autoimmune cytopenias
C063	F	48	Normal	Normal	Normal	Normal	↓ ↓	Normal	↓ ↓	No disease-related complications
C065	M	5	Normal	Normal	Normal	Normal	↓	↓	↓ ↓	No disease-related complications
C072	F	34	↓ ↓	Normal	Normal	Normal	↓ ↓	↓/↓↓	↓ ↓	No disease-related complications
C078	M	13	Normal	Normal	Normal	Normal	↓ ↓	Normal	↓ ↓	No disease-related complications
C082	F	11	↓ ↓	Normal	Normal	Normal	↓ ↓	↓ ↓	↓ ↓ b	Autoimmune cytopenias
C085	F	37	Normal	Normal	Normal	Normal	↓	↓ ↓	↓ ↓	No disease-related complications
C089	F	17	↓ ↓	Normal	Normal	Normal	↓ ↓	↓	↓ ↓	No disease-related complications
D038	M	22	Normal	Normal	Normal	Normal	↓ ↓	↓ ↓	↓ ↓	No disease-related complications
D209	M	41	Normal	Normal	Normal	Normal	↓ ↓	↓	↓ ↓	No disease-related complications
D232	F	44	↑	↑	↑	Normal	↓	Normal	↓ ↓	No disease-related complications
D269	F	44	Normal	↓	↓	Normal	↓ ↓	Normal	↓ ↓	Polyclonal lymphoproliferation; autoimmune cytopenias
D276	M	1	↓ ↓ ↓	Normal	Normal	Normal	↓ ↓	↓ ↓	↓ ↓	No disease-related complications
D325	F	35	Normal	↓	Normal	Normal	↓ ↓	↓	↓ ↓	Polyclonal lymphoproliferation; autoimmune cytopenias
D334	M	15	Normal	Normal	Normal	Normal	↓ ↓	↓	↓ ↓	No disease-related complications
D345	F	20	Normal	↑	Normal	↑	↓ ↓	↓	↓ ↓	No disease-related complications
D374	M	3	Normal	Normal	Normal	Normal	↓ ↓	↓ ↓	↓ ↓	No disease-related complications
D575	M	2	Normal	Normal	Normal	Normal	↓ ↓	↓ ↓	↓ ↓	No disease-related complications
D641	F	20	Normal	↓	↓	Normal	↓ ↓	↓/↓↓	↓ ↓	No disease-related complications
D667	M	13	Normal	Normal	Normal	Normal	↓ ↓	↓	↓ ↓	No disease-related complications
D705	F	49	Normal	Normal	Normal	Normal	↓/↓↓	↓/↓↓	Normal/↓	No disease-related complications
D745	M	5	Normal	↑	↑	↑	↓ ↓	Normal	↓ ↓	No disease-related complications
D765	F	30	Normal	Normal	Normal	Normal	↓ ↓	Normal	↓ ↓	No disease-related complications
D839	M	25	Normal	↓	Normal	Normal	↓ ↓	↓	↓ ↓	Polyclonal lymphoproliferation

↓ = between lower normal and half lower normal values; ↓↓ = less than half lower normal value; ↓↓↓ = undetectable; ↑ = between upper normal value and twice upper normal value;↑↑ = more than twice upper normal value.

a Values at diagnosis.

b Values at diagnosis not available; values listed are post-infusion of intravenous immunoglobulin, which does not alter IgA levels.

**Table 2:** Additional details on variants highlighted in the text.

Chromosome: position	Gene symbol	Transcript variant	Protein variant	dbSNP ID	Case samples with variant (freq)	1000 Genomes frequency	No. WGS500 (freq)	SIFT function prediction	SIFT score	PolyPhen-2 function prediction	Conservation phyloP p-value	Samples
*Described variants*												
17:16842991	*TNFRSF13B*	c.752C>T	p.P251L	34562254	7 (20.59)	18.07	47.0 (19.67)	Tolerated	0.69	Benign		C006, C028, C038, C072, D667, D705, D745
17:16843084	*TNFRSF13B*	c.659T>C	p.V220A	56063729	1 (2.94)	1.03	10.0 (4.18)	Tolerated	0.86	Benign		D269 (hom)
17:16843666	*TNFRSF13B*	c.605G>A	p.R202H	104894649	1 (2.94)		2.0 (0.84)	Damaging	0.02	Benign		D38
17:16852187	*TNFRSF13B*	c.310T>C	p.C104R	34557412	1 (2.94)	0.32	5.0 (2.09)	Damaging	0	Probably damaging	2.90E+00	D38
17:16852282	*TNFRSF13B*	c.215G>A	p.R72H	55916807	1 (2.94)	0.32	3.0 (1.26)	Tolerated	0.29	Benign		C003
19:54314003	*NLRP12*	c.910C>T	p.H304Y	141245482	1 (2.94)	0.27	1.0 (0.42)	Damaging	0.04	Probably damaging		C065
22: 42322716	*TNFRSF13C*	c.62C>G	p.P21R	77874543	2 (5.88)	5.59	22.0 (9.21)	Tolerated	0.64	Benign		C038, C089 (Hom)
*GWAS*												
6:30671229	*MDC1*	c.5648G>A	p.R1883Q	28994875	2 (5.88)	1.14	5 (2.09)	Damaging	0.03	Probably damaging	5.25E−04	C023, C078
6:30671726	*MDC1*	c.5234C>G	p.P1745R	28994871	5 (14.71)	1.37	9 (3.77)	Damaging	0.04	Probably damaging		C006, C024, D269 (hom), D667, D745
6:30673064	*MDC1*	c.3896G>A	p.R1299Q	144657716	5 (14.71)	3.60	9 (3.77)	Activating	1	Benign		C006, C024, D269 (hom), D667, D745
6:30673625	*MDC1*	c.3335C>T	p.S1112F	28987085	5 (14.71)	1.37	9 (3.77)	Damaging	0.01	Possibly damaging		C006, C024, D269 (hom), D667, D745
6:31598523	*PRRC2A*	c.2410C>T	p.R804C	11538262	1 (2.94)	0.74	7 (2.93)		Probably damaging		3.89E−03	C089
6:31599916	*PRRC2A*	c.3466C>T	p.R1156W	200579772	1 (2.94)					Probably damaging	6.38E−04	C036
6:31600639	*PRRC2A*	c.4189C>T	p.R1397W	201074309	1 (2.94)					Probably damaging	3.05E−03	D209
6:31605016	*PRRC2A*	c.6248T>C	p.F2083S	35595439	3 (8.82)	0.51	7 (2.93)			Possibly damaging	8.13E−04	C006, C024, D269 (hom)
6:31929737	*SKIV2L*	c.970C>T	p.R324W	36038685	1 (2.94)	1.10	4 (1.67)	Damaging	0.01	Possibly damaging		D641
6:31931903	*SKIV2L*	c.1860+1_1860+10delGTGCGTCTGT			1 (2.94)						5.46E−06	C044
*PID genes*												
2:220022915	*NHEJ1*	c.170G>A	p.R57Q	61753339	1 (2.94)		1.0 (0.42)	Damaging	0	Probably damaging	4.54E−02	D325
4:151392815*	*LRBA*	c.6661T>C; c.6628T>C	p.S2221P; p.S2210P		1 (2.94)			Damaging	0	Probably damaging	7.35E−04	D374
6:32803458	*TAP2*	c.701T>A	p.L234Q	138708621	1 (2.94)			Damaging	0.02	Probably damaging		D641
7:6045634	*PMS2*	c.52A>G	p.I18V	63750123	2 (5.88)	0.43	9.0 (3.77)	Damaging	0	Probably damaging	8.00E−03	C085, D374
8:42176165	*IKBKB*	c.1330C>T; c.1336C>T; c.1159C>T	p.R444W; p.R387W; p.R446W	202136671	1 (2.94)			Damaging	0.01	Possibly damaging	7.91E−01	D345
8:48691175	*PRKDC*	c.11698C>G; c.11605C>G	p.L3869V; p.L3900V	201214138	1 (2.94)					Probably damaging	3.27E+00	C024
8:48775035*	*PRKDC*	c.5818T>C	p.Y1940H		1 (2.94)					Probably damaging	1.31E−02	D232
10:14965056	*DCLRE1C*	c.640T>A; c.985T>A; c.625T>A	p.L214M; p.L329M; p.L209M	41299658	1 (2.94)	0.05	1.0 (0.42)	Damaging	0.01	Probably damaging		D334
11:4108092	*STIM1*	c.1859+1G>A; c.1541+319G>A		118128831	4 (11.76)	0.51	9.0 (3.77)				3.91E−03	C024, C044, D232, D269
11:36614561	*RAG2*	c.1158C>A	p.F386L	34629171	2 (5.88)	0.37	8.0 (3.35)	Damaging	0.02	Probably damaging		C019, C023
11:94178974*	*MRE11A*	c.1783+1411T>C; c.1867+2T>C			1 (2.94)						9.66E−01	C006
11:108143456	*ATM*	c.3161C>G	p.P1054R	1800057	1 (2.94)	1.51	11.0 (4.6)	Damaging	0.01	Probably damaging	1.33E−02	C082
12:25368405	*KRAS*	c.451-5560T>A; c.540T>A	p.C180*	373169526	1 (2.94)						8.51E+00	C089
12:133252342*	*POLE*	c.1085A>G	p.Y362C		1 (2.94)			Damaging	0	Probably damaging	7.41E−03	D839
16:27414502	*IL21R*	c.11G>A; c.−17+563G>A	p.R4H	117535117	2 (5.88)	0.65	4.0 (1.67)	Damaging	0			C028, D38
16:27460561	*IL21R-AS1; IL21R*	c.1574T>A; c.1640T>A	p.V525D; p.V547D	200674281	1 (2.94)			Damaging	0.04	Possibly damaging		D345
19:1627422	*TCF3*	c.302A>G	p.K101R	41275842	1 (2.94)	0.63	2.0 (0.84)	Damaging	0.01	Possibly damaging	5.18E+00	D374
19:42383644	*CD79A*	c.305C>A; c.419C>A	p.T102N; p.T140N	148797987	2 (5.88)	0.31	1.0 (0.42)	Damaging	0.02	Probably damaging	3.30E+00	C089, D575
19:55494283	*NLRP2*	c.1151C>G; c.1148C>G; c.1217C>G	p.T383R; p.T406R; p.T384R	139903547	3 (8.82)	0.34	2.0 (0.84)	Damaging	0.01	Probably damaging	3.52E−01	C024, C038, D38
X:100609671*	*BTK*	c.1049dupA; c.1577dupA; c.1679dupA	p.N350fs*11; p.N526fs*11; p.N560fs*11		1 (2.94)							D276 (hemi)
X:100611132*	*BTK*	c.1474C>T; c.1039-1450C>T; c.1576C>T	p.R492C; p.R526C		1 (2.94)			Damaging	0.05	Possibly damaging		C063
X:154005089*	*DKC1*	c.1495_1497delAAG; c.*718_*720delAAG; c.1510_1512delAAG	p.K505del; p.K500del		1 (2.94)		2.0 (0.84)					C019 (hemi)
*Most common variants*												
19:54756246	*LILRB5*	c.1556T>C; c.1259T>C; c.1559T>C	p.I420T; p.I519T; p.I520T	117421142	6 (19.35)	0.87	9 (3.77)	Activating	1	Benign		C003, C023, C038, D38, D334, D705
*1 hop genes*												
1:161599778	*FCGR3B*	c.214G>A; c.217G>A; c.109G>A; c.58G>A	p.V73M; p.V37M; p.V72M; p.V20M	375357751	1 (3.23)		0	Damaging	0	Probably damaging		C089
2:111923737	*BCL2L11*	c.*2124C>T; c.*2139C>T; c.*2014C>T; c.*2151C>T; c.*1929C>T; c.*2141C>T; c.*2195C>T; c.*2144C>T		144847549	1 (3.23)	0.61	(0)				9.10E−03	C032
2:234113242	*INPP5D*	c.3443C>G; c.3446C>G	p.A1149G; p.A1148G	375825105	1 (3.23)		0			Possibly damaging	2.90E−04	C065
4:2834080	*SH3BP2*	c.1513C>T; c.1600C>T; c.1429C>T	p.R534W; p.R477W; p.R505W	148761331	1 (3.23)	0.28	3 (1.26)	Damaging	0.01	Possibly damaging		C078
4:102751014	*BANK1*	c.120G>C; c.30G>C; c.71-25178G>C	p.W40C; p.W10C	35978636	2 (6.45)	0.55	8 (3.35)	Damaging	0	Probably damaging	4.55E−05	C032, C082
5:134086744	*CAMLG*	c.*104C>A		11552196	3 (9.68)	0.8	5 (2.09)					D374, D575, D705
5:150422217*	*TNIP1*	c.1018C>G; c.859C>G	p.Q287E; p.Q340E		1 (3.23)		0	Damaging	0.05	Probably damaging	3.30E−06	D38
5:150441774*	*TNIP1*	c.113-1G>T; c.272-1G>T			1 (3.23)		0				1.05E−03	D667
5:158124754*	*EBF1*	c.*1364_*1365insT			1 (3.23)		1 (0.42)				1.68E−06	C032
6:32823948	*PSMB9*	c.94G>A	p.V32I	241419	1 (3.23)	2.19	10 (4.18)	Damaging	0		7.98E−06	D209
6:32826267	*PSMB9*	c.517C>T	p.R173C	17213861	2 (6.45)	0.14	4 (1.67)	Damaging	0.01			C024, C036
6:138198245	*TNFAIP3*	c.838C>T	p.R280W	150198888	1 (3.23)	0.04	0	Damaging	0	Possibly damaging		C023
8:11418861*	*BLK*	c.1080C>A	p.D360E		1 (3.23)		0	Damaging	0	Possibly damaging	2.13E−04	C003
11:77937972	*GAB2*	c.746A>G; c.632A>G	p.Y249C; p.Y211C	146509778	1 (3.23)		1 (0.42)	Damaging	0.03	Possibly damaging	1.69E−05	C038
19:932497*	*ARID3A*	c.460_462delGAG	p.E154del		1 (3.23)		0					C072

The complete results of the different analyses can be found in Tables S3–S7. Variants are heterozygous unless the sample number is followed by (hom) which indicates a homozygous variant or (hemi) in which case the variant is hemizygous. Novel variants are marked with *. No. patients WGS500 indicates the number of patients in the WGS500 cohort (excluding CVID) with the variant. All variants have been confirmed in IGV.

**Table 3:** Overview of basic filtering applied in the majority of the analyses.

Filter	Thresholds	Rationale	Number of variants in 34 patients (number of genes)	Number of variants in 31 patients (number of genes)
			14,819,871 (20,891)	14,457,884 (20,627)
Confidence	Call quality is at least 20 in any sample Variant passed upstream pipeline filtering Read depth is at least 10 in any sample Variant is outside top 0.2% most exonically variable 100 base windows in healthy public genomes	Exclude poor quality variant calls	11,304,176 (20,594)	11,038,008 (20,577)
No. reverse reads	≥1 reads containing variant	Reduce strand bias	11,118,902 (20,583)	10,859,616 (20,564)
No. forward reads	≥1 reads containing variant	Reduce strand bias	10,940,142 (20,571)	10,687,781 (20,554)
Common variants	Variant has an allele frequency less than 5% in 1000 Genomes Project	Exclude common variants	4,808,176 (20,132)	4,582,532 (20,080)
WGS500 MAF	Variant has an allele frequency less than 5% in non-CVID, non-cancer WGS500 samples	Exclude technical artefacts	2,721,014 (19,678)	2,537,263 (19,584)
Predicted deleterious	Pathogenic/likely pathogenic or associated with any type of gain of function or associated with loss of function (frameshift, insertions/deletions, changes in start of stop codon, missense mutations which are predicted to not be tolerated by SIFT or PolyPhen, splice site loss up to 2 bp into the intron, deleterious to a micro RNA or variants leading to a structural variants)	Exclude variants which are unlikely to have effect on gene function	4768 (3737)	4422 (3524)

Details on the settings used and the rationale behind using these filters and the number of variants left after filtering in either 31 or 34 patients are indicated.

**Table 4:** Overlap of enriched pathways between WGS and RNA-seq. Overview of the 24 pathways found in both the 100 most significantly enriched pathways for WGS and the pathways significantly enriched for differentially expressed genes from the RNA-seq analysis.

Pathway	p value RNA-seq	p value WGS
Allograft rejection signalling	2.14E–03	4.74E–08
CD28 signalling in T helper cells	8.91E–03	1.01E–06
Haematopoiesis from pluripotent stem cells	1.82E–02	1.45E–06
Type I diabetes mellitus signalling	6.61E–03	3.90E–06
Graft-versus-host disease signalling	1.38E–02	7.66E–06
Autoimmune thyroid disease signalling	1.55E–04	8.86E–06
Dendritic cell maturation	2.69E–05	2.31E–05
Role of osteoblasts, osteoclasts and chondrocytes in rheumatoid arthritis	2.95E–03	3.83E–05
Altered T cell and B cell signalling in rheumatoid arthritis	1.45E–02	5.36E–05
iCOS–iCOSL signalling in T helper cells	2.95E–02	5.61E–05
Communication between innate and adaptive immune cells	3.24E–03	5.88E–05
Systemic lupus erythematosus signalling	1.12E–02	6.39E–05
Role of NFAT in regulation of the immune response	5.89E–04	8.62E–05
T helper cell differentiation	9.33E–04	2.47E–04
PKCθ, signalling in T lymphocytes	8.91E–03	8.02E–04
IL-6 signalling	3.80E–02	8.83E–04
Role of macrophages, fibroblasts and endothelial cells in rheumatoid arthritis	1.91E–02	1.06E–03
IL-10 signalling	8.71E–04	1.31E–03
Tec kinase signalling	7.41E–03	1.89E–03
Type II diabetes mellitus signalling	3.80E–02	2.28E–03
Death receptor signalling	2.57E–02	3.78E–03
LPS-stimulated MAPK signalling	4.57E–02	4.84E–03
PEDF signalling	4.27E–02	1.34E–02
RANK signalling in osteoclasts	1.51E–02	1.80E–02

## Abbreviations

BCR: (B-cell receptor)
CVIDs: (Common Variable Immunodeficiency Disorders)
ESID: (European Society for Immunodeficiencies)
GWAS: (Genome-wide Association Study)
HLA: (Human Leucocyte Antigen)
IPA: (Ingenuity Pathway Analysis)
IVA: (Ingenuity Variant Analysis)
LIR: (Leucocyte Ig receptor)
PDB: (Protein Data Bank)
PIDs: (Primary Immunodeficiency Disorders)
NHEJ: (Non-homologous End Joining)
RNA-seq: (RNA sequencing)
SNVs: (single nucleotide variants)
WGS: (whole genome sequencing)
XLA: (X-linked agammaglobulinaemia)
