# Supplementary material for: Application of whole genome and RNA sequencing to investigate the genomic landscape of common variable immunodeficiency disorders
Source: Clin Immunol. Author manuscript; Available in PMC 2015 Oct 12. (PMC4601528; doi:10.1016/j.clim.2015.05.020)
Supplement: Supplementary material [file NIHMS64345-supplement-Supplementary_material.doc]

**Supplementary notes**

**Note S1** List of described PID genes used for analysis of variants or differential expression of described PID genes.

*ACP5*

*ACT1*

*ACTB*

*ADA*

*ADAM17*

*ADAR1*

*AICDA*

*AIRE*

*AK2*

*AP3B*

*AP3B1*

*APOL1*

*ATM*

*BLM*

*BLNK*

*BTK*

*C/EBPE*

*C16orf57*

*C1QA*

*C1QB*

*C1QC*

*C1R*

*C1S*

*C2*

*C3*

*C4A*

*C4B*

*C5*

*C6*

*C7*

*C8A*

*C8B*

*C9*

*CARD11*

*CARD14*

*CARD9*

*CASP10*

*CASP8*

*CD3D*

*CD3E*

*CD3G*

*CD3Z*

*CD40*

*CD40L*

*CD46*

*CD79A*

*CD79B*

*CD8A*

*CFB*

*CFD*

*CFH*

*CFHR1*

*CFHR2*

*CFHR3*

*CFHR4*

*CFHR5*

*CFI*

*CFP*

*CHD7*

*CIAS1*

*CIITA*

*COLEC11*

*CORO1A*

*CSF2RA*

*CTSC*

*CXCR4*

*CYBA*

*CYBB*

*DCLRE1C*

*DKC1*

*DNMT3B*

*DOCK8*

*ELANE*

*EVER1*

*EVER2*

*FADD*

*FCN3*

*FERMT3*

*FOXN1*

*FOXP3*

*FPR1*

*G6PC3*

*G6PD*

*G6PT1*

*GATA2*

*GFI1*

*HAX1*

*HOIL 1*

*IFNGR1*

*IFNGR2*

*IGHM*

*IGKC*

*IGLL1*

*IKBA*

*IKBKB*

*IKBKG*

*IKZF1*

*IL10*

*IL10RA*

*IL10RB*

*IL12B*

*IL12RB1*

*IL17F*

*IL17RA*

*IL1RN*

*IL21R*

*IL2RA*

*IL2RG*

*IL36RN*

*IL7RA*

*IRAK4*

*IRF8*

*ISG15*

*ITCH*

*ITGB2*

*ITK*

*ITK*

*JAK3*

*KRAS*

*LCK*

*LIG4*

*LPIN2*

*LYST*

*MAGT1*

*MALT1*

*MASP1*

*MASP2*

*MBL2*

*MCM4*

*MEFV*

*MIRL*

*MLL2*

*MRE11*

*MSH5*

*MTHFD1*

*MVK*

*MYD88*

*NBS1*

*NCF1*

*NCF2*

*NCF4*

*NHEJ1*

*NHP2*

*NKX2-5*

*NLRP3*

*NOD2*

*NOLA3*

*NRAS*

*ORAI1*

*OX40*

*PFC*

*PIGA*

*PIK3R1*

*PLDN*

*PMS2*

*PNP*

*POLE1*

*PRF1*

*PRKCD*

*PRKDC*

*PSMB8*

*PSTPIP1*

*PTPRC*

*RAB27a*

*RAC2*

*RAG1*

*RAG2*

*RFX5*

*RFXANK*

*RFXAP*

*RHOH*

*RMRP*

*RNASEH2A*

*RNASEH2B*

*RNASEH2C*

*RNF168*

*ROBLD3*

*RPSA*

*RTEL1*

*SAMHD1*

*SBDS*

*SEMA3E*

*SERPING1*

*SH2D1A*

*SH3BP2*

*SLC29A3*

*SLC35C1*

*SLC46A1*

*SMARCAL1*

*SP110*

*SPINK5*

*STAT1*

*STAT2*

*STAT3*

*STAT5B*

*STIM1*

*STK4*

*STX11*

*STXBP2*

*TAP1*

*TAP2*

*TAPBP*

*TAZ*

*TBK1*

*TBX1*

*TCF3*

*TCN2*

*TERC*

*TERT*

*THBD*

*TINF2*

*TLR3*

*TNFRSF1A*

*TNFRSF6*

*TNFSF6*

*TRAC*

*TRAF3*

*TREX1*

*TRIF*

*TTC7A*

*TWEAK*

*TYK2*

*UNC119*

*UNC13D*

*UNC93B1*

*UNG*

*VPS13B*

*VPS45*

*WAS*

*WIPF1*

*XIAP*

*ZAP70*

*ZBTB*

**Note S2** Additional details on analysis of the grandmother grandson pair.

Patient D232 is the maternal grandmother of patient C018 (male). The mother of C018 has been diagnosed with IgA deficiency (IgAD). To identify variants of interest in this family, we filtered variants in both patients as described in Table 3. This resulted in 36 shared heterozygous variants and one shared homozygous variant (Table S15). Interestingly, the homozygous variant is found in the mitochondrial gene *MT-CO2*. This would fit with previous studies suggesting a shared pathogenesis between IgAD and CVID and predominance of maternal transmission of familial IgAD[72]. This is an attractive hypothesis but further functional analysis needs to be undertaken to investigate this further.

**Note S3** List of genes associated with CVID in literature as used for the ‘1 hop analysis’.

*CD19*

*CD81*

*ICOS*

*MS4A1*

*TACI*

*TNFRSF13C*

*CD21*

*CD27*

*PLCG2*

*LRBA*

*PIK3CD*

*NFKB2*

*NLRP12*

*FCGR2A*

**
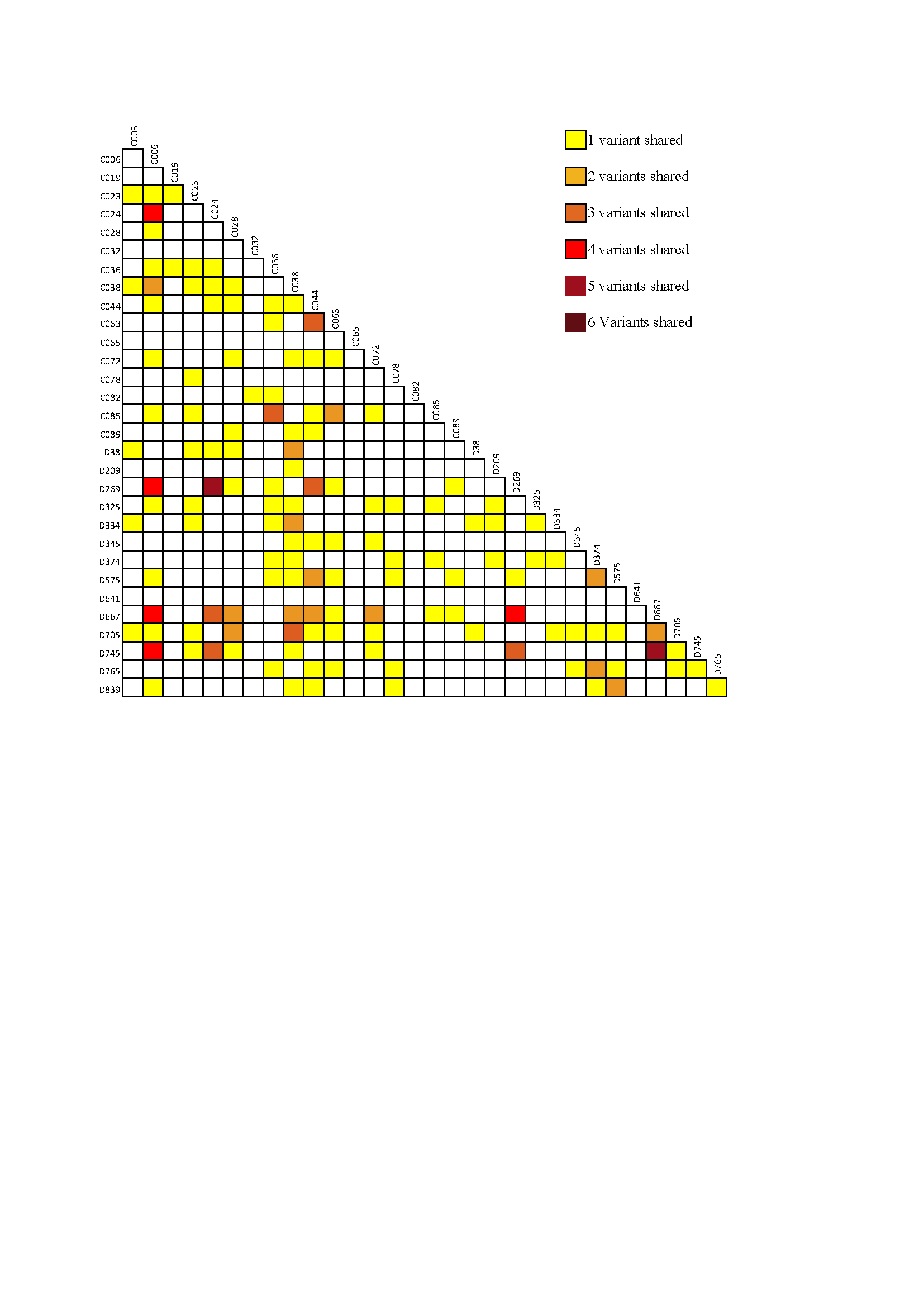
**

**Supplementary figure 1** Distribution of variants between patients. **(a)** The number of variants from Supplementary Table 3-7 online overlapping between patients.

**Supplementary figure 2** The distribution of variants (small nodes) listed in Supplementary table 3-7 between patients (big nodes). The colour of the big nodes corresponds to the clinical phenotype of the patient.

**Supplementary figure 3** The distribution of genes (small nodes) listed in Supplementary table 3-7 between patients (big nodes). The colour of the big nodes corresponds to the clinical phenotype of the patient.

**Supplementary figure 4** Quality control for RNA-seq. **(a)** Alignment of reads to different regions of the genome. **(b)** Hierarchical tree illustrating the relationship between clusters of samples based on reads which map to genes. The height of the branches indicates the strength of separation between individuals.

**Supplementary figure 5** Differential expression of PID-associated genes in CVID patients and healthy controls.

**Supplementary figure 6** Expression of variant-containing genes with evidence of altered expression in CVID patients. The red dots represent the patient with a variant in a gene, which also shows evidence of altered gene expression compared to healthy controls. **(a)** Genes with increased expression in CVID patients (fold change >2). **(b)** Genes with decreased expression in CVID patients (fold change <0.5)

**WGS500 Consortium: names and affiliations of authors**

**Steering Committee:** Peter Donnelly (Chair)^1^, John Bell^2^, David Bentley^3^, Gil McVean^1^, Peter Ratcliffe^1^, Jenny Taylor^1,4^, Andrew Wilkie^4,5^

**Operations Committee:** Peter Donnelly^1^ (Chair), John Broxholme^1^, David Buck^1^, Jean-Baptiste Cazier^1^, Richard Cornall^1^, Lorna Gregory^1^, Julian Knight^1^, Gerton Lunter^1^, Gil McVean^1^, Jenny Taylor^1,4^, Ian Tomlinson^1,4^, Andrew Wilkie^4,5^

**Sequencing & Experimental Follow up:** David Buck^1^ (Lead), Christopher Allan^1^, Moustafa Attar^1^, Angie Green^1^, Lorna Gregory^1^, Sean Humphray^3^, Zoya Kingsbury^3^, Sarah Lamble^1^, Lorne Lonie^1^, Alistair Pagnamenta^1^, Paolo Piazza^1^, Guadelupe Polanco^1^, Amy Trebes^1^

**Data Analysis:** Gil McVean^1^ (Lead), Peter Donnelly^1^, Jean-Baptiste Cazier^1^, John Broxholme^1^, Richard Copley^1^, Simon Fiddy^1^, Russell Grocock^3^, Edouard Hatton^1^, Chris Holmes^1^, Linda Hughes^1^, Peter Humburg^1^, Alexander Kanapin^1^, Stefano Lise^1^, Gerton Lunter^1^, Hilary Martin^1^, Lisa Murray^3^, Davis McCarthy^1^, Andy Rimmer^1^, Natasha Sahgal^1^, Ben Wright^1^, Chris Yau^6^

1. The Wellcome Trust Centre for Human Genetics, Roosevelt Drive, Oxford, OX3 7BN, UK

2. Office of the Regius Professor of Medicine, Richard Doll Building, Roosevelt Drive, Oxford, OX3 7LF, UK

3. Illumina Cambridge Ltd., Chesterford Research Park, Little Chesterford, Essex, CB10 1XL, UK

4. NIHR Oxford Biomedical Research Centre, Oxford, UK

5. Weatherall Institute of Molecular Medicine, John Radcliffe Hospital, Headington, Oxford OX3 9DS, UK

6. Imperial College London, South Kensington Campus, London, SW7 2AZ, UK

**References**

[72] I. Vorechovsky, A.D. Webster, A. Plebani, and L. Hammarstrom, Genetic linkage of IgA deficiency to the major histocompatibility complex: evidence for allele segregation distortion, parent-of-origin penetrance differences, and the role of anti-IgA antibodies in disease predisposition, Am J Hum Genet 64 (1999)1096-1109.
